# Targeted reactivation of the novel tumor suppressor DAPK1, an upstream regulator of p53, in high‐grade serous ovarian cancer by mRNA liposomes reduces viability and enhances drug sensitivity in preclinical models

**DOI:** 10.1002/cac2.70029

**Published:** 2025-05-20

**Authors:** Monika Raab, Balázs Győrffy, Samuel Peña‐Llopis, Daniela Fietz, Monika Kressin, Margareta Kolaric, Matthias Ebert, Khayal Gasimli, Sven Becker, Mourad Sanhaji, Klaus Strebhardt

**Affiliations:** ^1^ Department of Gynecology Medical School, Goethe University Frankfurt (Main) Germany; ^2^ Department of Bioinformatics and Department of Pediatrics Semmelweis University Budapest Hungary; ^3^ Department of Biophysics Medical School, University of Pecs Pecs Hungary; ^4^ HUN‐REN TTK Cancer Biomarker Research Group Budapest Hungary; ^5^ Translational Genomics, Department of Ophthalmology University Hospital Essen Essen Germany; ^6^ German Cancer Consortium (DKTK) at University Hospital Essen and German Cancer Research Center (DKFZ) Heidelberg Germany; ^7^ Institute for Veterinary Anatomy, Histology and Embryology Giessen Germany; ^8^ Georg‐Speyer‐Haus, Goethe University Frankfurt Germany; ^9^ German Cancer Consortium (DKTK) at Goethe University Frankfurt and German Cancer Research Center (DKFZ) Heidelberg Germany

**List of abbreviations**
HGSOC: high‐grade serous ovarian cancer
DAPK1: death‐associated protein kinase 1
TP53: tumor protein 53
IVT‐mRNA: in vitro transcribed‐messenger RNA
KD‐AR‐DD: kinase‐ankyrin repeats‐and death‐domain
KD‐AR‐ROC‐COR‐DD: kinase‐,‐ankyrin repeats‐, ras of complex‐, C‐terminal of ROC‐, and death‐ domain
KD‐AR‐ROC‐DD: kinase‐,‐ankyrin repeats‐, ras of complex‐and death‐ domain
KD‐COR‐DD: kinase‐,‐C‐terminal of ROC‐, and death‐domain
KD‐DD: kinase‐ and‐death domain
KD: kinase domain
OVCAR‐3/‐4‐5/‐8‐PTX: ovarian cancer cell lines with “PTX” indicating paclitaxel resistance.
p14ARF: alternate reading frame protein
p21 (CIP1/WAF1): cyclin‐dependent kinase inhibitor p21
p16 (INK4a): cyclin‐dependent kinase inhibitor that inhibits CDK4/6
Bax: Bcl‐2‐associated X protein
p53‐pS20: p53 phospho‐Serine 20
PLK1: polo‐like kinase 1
Aurora A: Aurora kinase A
CDK1: cyclin‐dependent kinase 1
PARP: poly (ADP‐Ribose) polymerase
FAS: fas cell surface death receptor
Puma: p53 upregulated modulator of apoptosis
Noxa: pro‐apoptotic protein regulated by p53
Luc: Luciferase
HUVEC: human umbilical vein endothelial cells
IVIS: in vivo imaging system
pBeclin: phospho‐Beclin pThr 119
MCL2: myosin‐light chain 2
MDM2: murine double minute 2

1

Ovarian cancer, particularly high‐grade serous ovarian cancer (HGSOC), remains the most lethal gynecological malignancy, with a 5‐year survival rate of around 40% due to late diagnosis, recurrence, and the development of chemoresistance [1, 2]. Mutations in tumor protein 53 (*TP53*) occur in over 96% of HGSOC cases, impairing its tumor‐suppressive functions, including cell cycle control, DNA repair, and apoptosis. Mutant *TP53* promotes tumor progression, genomic instability, and resistance to standard therapies, thereby worsening patient outcomes [3, 4]. Death‐associated protein kinase 1 (*DAPK1*) is a key regulator of apoptosis and autophagy [5, 6]. While p53 can upregulate *DAPK1* expression, DAPK1 in turn stabilizes p53 by inhibiting its negative regulator, murine double minute 2 (MDM2). This reciprocal regulation forms a feedback loop that reinforces p53's tumor‐suppressive function. We identified aberrant DAPK1 expression in ovarian cancer and sought to investigate whether restoring DAPK1 function could serve as a potential therapeutic strategy. Recent advancements in mRNA‐based therapies offer a promising approach to gene restoration. Thus, we investigated whether in vitro‐transcribed (IVT)‐mRNA encoding DAPK1 could serve as an effective therapeutic strategy for HGSOC. Here, we explore the potential of mRNA‐based reactivation of DAPK1 to regulate cell survival and apoptosis in HGSOC.

In studies using mammalian vectors to deliver functional proteins for replacement therapy, reducing the length of recombinant DNA vectors has been shown to enhance transfection efficiency, translation, and persistence in cells [7, 8, 9]. Given the relatively long open reading frame of *DAPK1* (4,290 base pairs), we generated a series of constructs containing different functional domains of DAPK1 and assessed their anti‐tumor efficacy in ovarian cancer cells. We found that a truncated *DAPK1* variant, containing the kinase domain, ankyrin repeats, and death domain (KD‐AR‐DD), retained potent tumor‐suppressive activity despite being approximately 50% shorter than the wild‐type protein. Compared to other truncated constructs, mammalian vector‐based expression of KD‐AR‐DD strongly activated Caspase‐3/7 and significantly sensitized OVCAR‐3 cells to paclitaxel treatment (Supplementary Figure S1A‐C). Based on these findings, we selected KD‐AR‐DD as the basis for designing an IVT‐mRNA construct, referred to as ∆DAPK1‐mRNA (Figure 1A), optimized to induce cell death in ovarian cancer cells. For IVT‐mRNA synthesis, we employed a bacterial vector containing a T7 RNA polymerase promoter to drive transcription of human truncated *DAPK1*, focusing on optimizing translational efficacy and mRNA stability [9]. To deliver ∆DAPK1‐mRNA to HGSOC cells, we utilized a liposomal system with Lipofectamine MessengerMAX Transfection Reagent. Treatment of OVCAR‐8 cells with increasing concentrations of ∆DAPK1‐mRNA suppressed proliferative activity, as evidenced by reduced colony formation (Supplementary Figure S2A) and the downregulation of key cell cycle regulators, including polo‐like kinase 1 (PLK1), cyclin A/B, Aurora kinase A (Aurora A), and cyclin‐dependent kinase 1 (CDK1) (Supplementary Figure S2B). Furthermore, ∆DAPK1 phosphorylated classical targets of full‐length *DAPK1*, as shown in a titration experiment, including p53 at Ser20 and Beclin at Thr119, leading to p53 stabilization and promoting p53‐dependent apoptosis and autophagy, respectively (Supplementary Figure S2B, upper and lower panels). Additionally, ∆DAPK1 upregulated p14ARF expression, further contributing to p53 stabilization. Increased levels of apoptotic markers, such as cleaved poly (ADP‐Ribose) polymerase (PARP) and Caspase‐3, along with elevated Caspase‐3/7 activity, further confirmed the pro‐apoptotic effects of ∆DAPK1 expression (Supplementary Figure S2B‐C).

**FIGURE 1:**
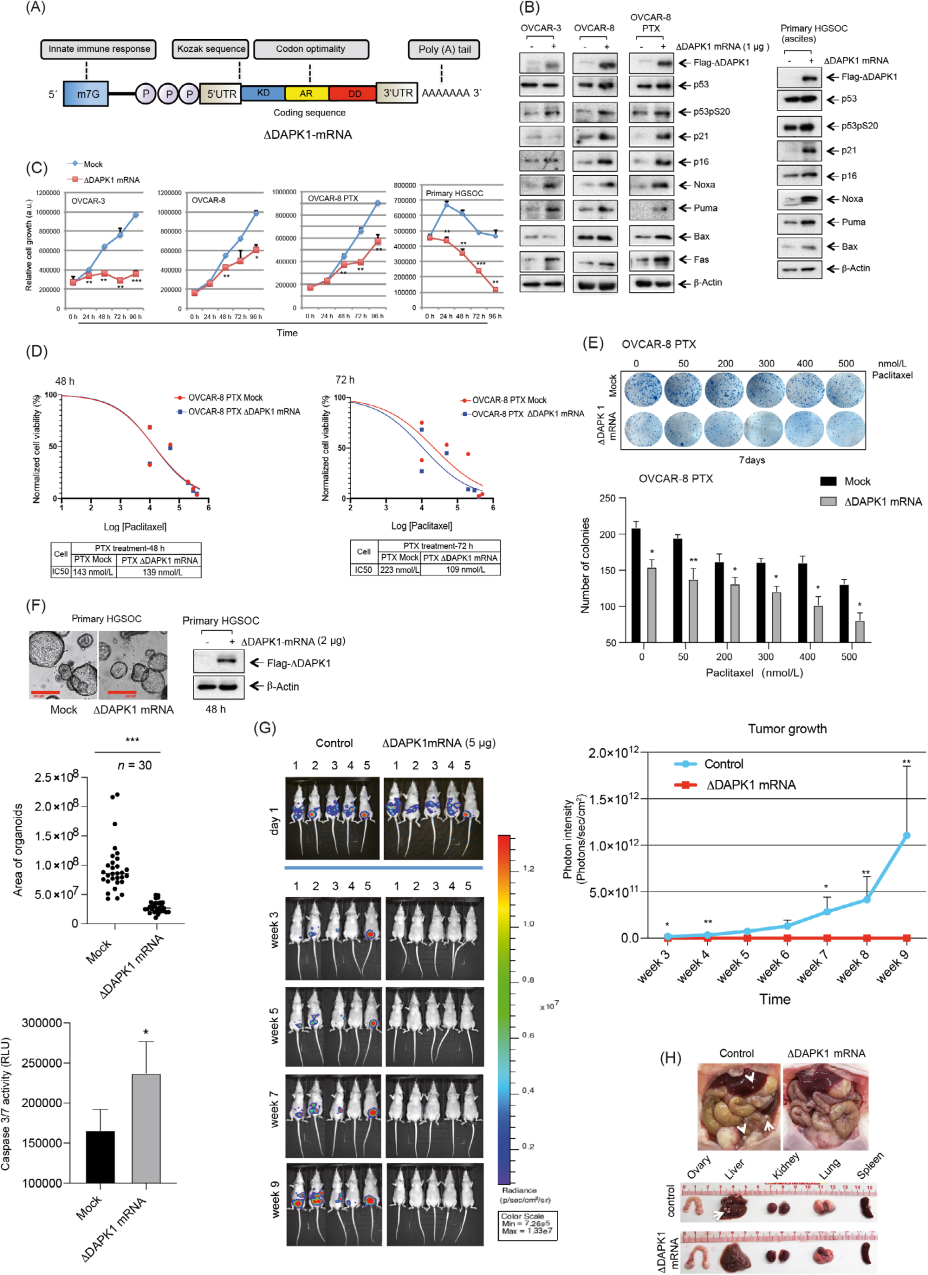
Functional characterization and therapeutic potential of ΔDAPK1‐IVT mRNA in high‐grade serous ovarian cancer models. (A) Key structural elements of ΔDAPK1‐IVT mRNA used in this study. Modifications such as the addition of synthetic cap analogs at the 5’‐end, a poly(A) tail at the 3’‐end, and modified nucleosides (e.g., 5‐methylcytidine, pseudouridine) enhance the stability and translational activity of synthetic mRNAs while reducing their immunogenicity. (B) HGSOC cell lines (OVCAR‐3, ‐8), a paclitaxel‐resistant cell line (OVCAR‐8 PTX), and HGSOC ascites‐derived cells were transfected with 1 µg ΔDAPK1‐IVT mRNA. WB analysis of cell lysates was performed using antibodies against Flag, p53, phosphorylated p53 (p53pS20), p21, p16, Noxa, Puma, Bax, Fas, and β‐Actin. (C) HGSOC cell lines and primary HGSOC cells were mock‐transfected or transfected with 1 µg ∆DAPK1‐mRNA, and proliferation was measured using the CellTiter‐Blue Cell Viability assay over 96 hours. * *P* < 0.05, ** *P* < 0.01, *** *P* < 0.001, unpaired two‐tailed Student's t‐test. (D) OVCAR‐8‐PTX (mock or ΔDAPK1‐IVT mRNA‐transfected) were treated with increasing concentrations of paclitaxel. Cell viability was measured using the CellTiter‐Blue assay at 48 hours (left panel) and 72 hours (right panel). Proliferation rates were normalized to DMSO‐treated controls (vehicle control  =  0 on the x‐axis), and IC50 values were calculated for each condition. (E) OVCAR‐8‐PTX cells were transfected with increasing concentrations of ΔDAPK1‐IVT mRNA. On day 7, the number of 3D colonies was quantified. Representative images (upper panel) and a dose‐dependent distribution of colonies (lower panel; n = 3) are shown. * P < 0.05, ** P < 0.01; unpaired two‐tailed Student's t‐test. (F) Representative images of organoids transfected with 2 µg ΔDAPK1‐IVT mRNA on day 2 (upper left panel). Scale bar = 300 µm. WB analysis of mock‐ and ΔDAPK1‐IVT mRNA‐transfected HGSOC organoid lysates using Flag and β‐actin antibodies (upper right panel). Organoid size was measured using ImageJ (middle panel; *n* = 30). *** *P* < 0.001; unpaired two‐tailed student's t‐test. Caspase‐3/7 activity was assessed using the Caspase‐Glo 3/7 assay in mock‐ and ΔDAPK1‐IVT mRNA‐transfected HGSOC organoids (lower panel; *n* = 3). * *P* < 0.05; unpaired two‐tailed Student's t‐test. (G) Bioluminescence imaging of nude mice bearing metastatic, luciferase‐expressing OVCAR‐8 cells after liposomal ΔDAPK1‐IVT mRNA therapy (left panel). Mice received intraperitoneal injection of 2 × 10^6^ OVCAR‐8‐Luc cells, followed by twice‐weekly intraperitoneal injections of 5 µg liposomal ΔDAPK1‐IVT mRNA for 3 weeks, starting 24 hours post‐cell injection. Tumor burden was monitored weekly over 9 weeks using bioluminescence imaging (right panel; *n* = 8). The color scale represents photon flux (P/sec/cm2/steradian). The Mann‐Whitney U test was performed to assess differences between groups for each week. Statistically significant differences are indicated as follows: week 3 (*P* = 0.031), week 4 (*P* = 0.001), week 7 (*P* = 0.010), week 8 (*P* = 0.007), week 9 (*P* = 0.004). (H) Following laparotomy, peritoneal tissues and organs were examined for tumor dissemination. Tumor burden and organ sizes were assessed in each mouse. The arrowheads in white indicate metastatic nodules in the control mice. Abbreviations: KD‐AR‐DD, DAPK1 kinase domain, ankyrin repeats, death domain; ∆DAPK1‐mRNA, truncated form of DAPK1 (KD‐AR‐DD); p53, tumor protein p53; p53pS20, p53 phosphorylated at Ser20; p21 (CIP1/WAF1), cyclin‐dependent kinase inhibitor p21; p16 (INK4a), cyclin‐dependent kinase inhibitor that inhibits CDK4/6; Noxa, pro‐apoptotic protein regulated by p53; Puma, p53 upregulated modulator of apoptosis; Bax, Bcl‐2‐associated X protein; Fas, fas cell surface death receptor; OVCAR‐8 PTX, ovarian cancer cell lines with “PTX” indicating Paclitaxel resistance; HGSOC, high‐grade serous ovarian cancer; mRNA, messenger RNA, (a.u.) arbitrary units; WB, Western blot; Bax, Bcl‐2‐associated X protein.

Stabilization of p53 following ∆DAPK1‐mRNA expression led to the upregulation of its classical targets, including p21, p53 upregulated modulator of apoptosis (Puma), pro‐apoptotic protein regulated by p53 (Noxa), and fas cell surface death receptor (FAS), in OVCAR‐8, OVCAR‐8 PTX (a paclitaxel‐resistant cell line), and primary cells derived from patient tumor ascites. Ascitic fluid, which is associated with metastasis, is frequently encountered in advanced ovarian cancer and may contribute to disease relapse following chemotherapy [10] (Figure 1B, Supplementary Figure S1C). The observed increase of p53 target gene expression suggests a reactivation of p53 signaling and a p53‐dependent apoptotic pathway upon ∆DAPK1‐mRNA expression. Moreover, transfection with ∆DAPK1‐mRNA significantly inhibited cellular proliferation in OVCAR‐3, OVCAR‐8, paclitaxel‐resistant OVCAR‐8‐PTX, and primary HGSOC cells (Figure 1C).

To further assess the efficacy of ∆DAPK1‐mRNA, we tested its effects on additional HGSOC cell lines (OVCAR‐3, ‐4, ‐5, ‐8). While OVACR‐3, ‐4, ‐8, and the ascitic patient‐derived sample harbor *TP53* mutations, OVCAR‐5 is a *TP53*‐wild‐type cell line. We observed increased markers of cell death, including elevated Caspase‐3/7 activity, in response to ∆DAPK1‐mRNA treatment (Supplementary Figure S3A). While normal human cells (fibroblasts and HUVECs) exhibited only minimal PARP cleavage, OVCAR‐8 cells showed strong levels (Supplementary Figure S3B). These results indicate that ∆DAPK1‐mRNA selectively induces cell death in HGSOC cell lines while sparing normal cells.

Next, we evaluated whether ∆DAPK1‐mRNA could enhance the response of ovarian cancer cells to paclitaxel‐based standard therapy. ∆DAPK1‐mRNA expression significantly increased the sensitivity of paclitaxel‐resistant OVCAR‐8 PTX cells to paclitaxel (Figure 1D). A clonogenic assay further confirmed the sensitizing effect of ∆DAPK1‐mRNA, as OVCAR‐8 PTX cells treated with ∆DAPK1‐mRNA exhibited reduced colony formation upon paclitaxel treatment (Figure 1E).

We compared the effects of ∆DAPK1‐mRNA transfection in primary normal ovarian cells and primary HGSOC cells to further assess the therapeutic potential of restoring DAPK1 expression in a preclinical setting. Despite identical transfection levels (1 µg ∆DAPK1‐mRNA), initial comparisons between HGSOC cells and matched normal ovarian tissue from the same patient showed significantly higher Caspase‐3/7 activity in tumor cells (Supplementary Figure S4). Additionally, analysis of various organoids obtained from HGSOC patients also demonstrated that, under 3D cell culture conditions, ∆DAPK1‐mRNA therapy led to a marked reduction in primary HGSOC cell viability, as indicated by decreased organoid volume and increased Caspase‐3/7 activity (Figure 1F).

To further evaluate the clinical relevance of ∆DAPK1‐mRNA in metastatic HGSOC, we investigated whether liposomal ∆DAPK1‐mRNA could effectively target dispersed tumor cells in the peritoneal cavity of a xenograft mouse model. We intraperitoneally injected 2 × 10^6^ stable Luciferase (Luc)‐expressing OVCAR‐8 cells (OVCAR‐8/Luc cells), followed by intraperitoneal delivery of ∆DAPK1‐mRNA 1 day later. For 3 weeks, mice received twice‐weekly intraperitoneal injections of either ∆DAPK1‐mRNA (0.16 mg/kg) or a control. Remarkably, ∆DAPK1‐mRNA therapy completely inhibited tumor cell growth (Figure 1G). Throughout the observation period, body weight development remained comparable between both treatment groups, indicating no significant toxicity (Supplementary Figure S5).

Gross anatomical examination revealed extensive tumor masses in control mice, primarily on the peritoneal surfaces, adipose tissues, intestines, and omentum, with strong in vivo imaging system (IVIS) signals. In contrast, ΔDAPK1‐mRNA‐treated mice showed no visible tumors, maintained normal organ morphology, and showed no detectable tumor signals on IVIS imaging, indicating successful inhibition of tumor dissemination (Figure 1H, Supplementary Figure S6)

In conclusion, our findings demonstrate that a novel mRNA‐based approach can effectively restore DAPK1 expression in HGSOC. By designing and delivering a truncated, catalytically active form of DAPK1 (∆DAPK1‐mRNA), we successfully reactivated its pro‐apoptotic functions in HGSOC cells, including paclitaxel‐resistant models and primary patient‐derived tumor cells. Notably, the ability of ∆DAPK1‐mRNA to sensitize paclitaxel‐resistant HGSOC cells to chemotherapy highlights its potential to overcome chemoresistance. In vivo studies further confirmed that intraperitoneal administration of liposome‐delivered ∆DAPK1‐mRNA efficiently suppressed tumor growth, prevented peritoneal dissemination, and exhibited no apparent toxicity. These findings highlight mRNA‐based reactivation of *DAPK1* as a promising therapeutic strategy for HGSOC, targeting both tumor proliferation and chemoresistance. Moreover, they support further investigation of mRNA‐based gene restoration therapies as a viable approach for ovarian cancer treatment.

## AUTHOR CONTRIBUTIONS

Monika Raab and Klaus Strebhardt conceptualized and coordinated the study. Monika Raab, Mourad Sanhaji, and Klaus Strebhardt wrote the manuscript. Monika Raab, Khayal Gasimli, Matthias Ebert, Margareta Kolaric, Samuel Peña‐Llopis, Daniela Fietz, Monika Kressin, Mourad Sanhaji, and Klaus Strebhardt designed and performed experiments, interpreted results, and prepared figures. All authors read and approved the final version of the manuscript.

## CONFLICT OF INTEREST STATEMENT

The authors declare no potential conflicts of interest.

## FUNDING INFORMATION

This work was supported by grants from Deutsche Krebshilfe (70116875), and the German Cancer Consortium (DKTK, Heidelberg).

## ETHICS APPROVAL AND CONSENT TO PARTICIPATE

All tissues were obtained from patients who had underwent surgery at the University Hospital of Goethe University, with patient consent and approval from the Goethe University Committee for Ethical Review of Research involving Human Subjects (approval number: SGO‐1‐2017). All patients provided written informed consent. All animal experiments were approved by the regional council (Darmstadt) (V 54‐19c 18‐FK/1128).

## Supporting information



Supporting information

## Data Availability

The data generated in this study are available within this article and its Supplementary Materials.
