# Supplementary material for: Targeted reactivation of the novel tumor suppressor DAPK1, an upstream regulator of p53, in high‐grade serous ovarian cancer by mRNA liposomes reduces viability and enhances drug sensitivity in preclinical models
Source: Cancer Commun (Lond). 2025 May 20;45(8):966–70. doi: 10.1002/cac2.70029 (PMC12365538; doi:10.1002/cac2.70029)
Supplement: Supplementary file 1 — Supporting information [file CAC2-45-966-s001.docx]

**Supplementary Materials**

**Targeted reactivation of the novel tumor suppressor DAPK1, an upstream regulator of p53, in high-grade serous ovarian cancer by mRNA liposomes reduces viability and enhances drug sensitivity in preclinical models**

### Monika Raab^1^, Balázs Győrffy^2,3,4^, Samuel Peña-Llopis^5,6^, Daniela Fietz^7^, Monika Kressin^7^, Margareta Kolaric^8^, Matthias Ebert^8^, Khayal Gasimli^1^, Sven Becker^1^, Mourad Sanhaji^1,*^, Klaus Strebhardt^1,9,*^

^1^Department of Gynecology, Medical School, Goethe University, Frankfurt (Main), Germany.

^2^Department of Bioinformatics and Department of Pediatrics, Semmelweis University, Budapest, Hungary.

^3^Department of Biophysics, Medical School, University of Pecs, Pecs, Hungary.

^4^HUN-REN TTK Cancer Biomarker Research Group, Budapest, Hungary.

^5^Translational Genomics in Solid Tumors, Department of Ophthalmology, University Hospital, Essen, Germany.

^6^German Cancer Consortium (DKTK), Essen, Germany.

^7^Institute for Veterinary Anatomy, Histology and Embryology, Giessen, Germany.

^8^Georg-Speyer-Haus, Goethe University, Frankfurt, Germany.

^9^German Cancer Research Center (DKFZ), Heidelberg, Germany.

^*^Corresponding author:

Klaus Strebhardt; Department of Gynecology, Medical School, Goethe University, 60590 Theodor-Stern-Kai 7-9, Frankfurt (Main), Germany; Email: [strebhardt@em.uni-frankfurt.de](mailto:strebhardt@em.uni-frankfurt.de).

Mourad Sanhaji; Department of Gynecology, Medical School, Goethe University, 60590 Theodor-Stern-Kai 7-9, Frankfurt (Main), Germany; Email: Sanhaji@med.uni-frankfurt.de.

**Supplementary Materials and Methods**

**Generation of mRNA**

Using the reverse primer to add a 120A tail and restore the functional T7 promoter sequence, the plasmid template containing the truncated open reading frame of Flag-DAPK1 was amplified via PCR. These structures were capped (Cap 1) with CleanCap® AG (Tebu-Bio, Offenbach, Hessen, Germany), in which Pseudo-U completely replaced uridine-5′-triphosphate. After in vitro transcription (IVT), DNase (Tebu-Bio, Offenbach, Hessen, Germany) was employed to eliminate any remaining DNA template. For thorough mRNA purification, a spin column with a silica membrane (Tebu-Bio, Offenbach, Hessen, Germany) was used to remove the digested template, excess nucleoside triphosphates (NTPs), salts, and unnecessary capping analogs. DNase was also utilized to degrade residual DNA template. Additional purification steps using a Qiagen RNeasy (silica membrane) spin column removed excess NTPs, salts, and unneeded capping analogs. To eliminate any residual immunogenic 5′ triphosphate, the remaining uncapped material was treated with Antarctic phosphatase (Tebu-Bio, Offenbach, Hessen, Germany), followed by a second RNeasy spin column purification to extract the phosphatase. Purified mRNA was obtained from TriLink (Tebu-Bio, Offenbach, Hessen, Germany).

**Patients and tissue samples**

This study followed the "REporting recommendations for tumor MARKer prognostic studies" [30]. Ovarian carcinoma samples were collected from patients diagnosed with epithelial ovarian cancer at the Department of Gynecology, Goethe University Hospital, Frankfurt am Main, Germany, between January 2015 and December 2022, after informed consent was acquired. Three pathologists independently assessed the samples, and patients with different tumor types were excluded based on predetermined criteria. We examined samples from eight patients who underwent surgical resection to establish primary patient-derived ovarian cancer (OC) cell cultures. For cases with confirmed diagnoses, sufficient archival material was available for cell culture studies. The research involving human tissue was authorized by the local research ethics council (permission number: SGO-1-2017), and samples were handled anonymously.

**Cell lines, primary cells, and transfection**

The human ovarian cancer cell lines SKOV3, OVCAR-3, OVCAR-4, OVCAR-5, and OVCAR-8 were purchased from the DCTD Tumor Repository (Bethesda, MD, USA) and maintained in RPMI 1640 medium (#71870036, Thermo Fisher Scientific, Dreieich, Hessen, Germany) supplemented with 1% penicillin-streptomycin and 10% fetal bovine serum (FBS; #A5256701, Thermo Fisher Scientific, Dreieich, Germany). All cell lines were cultivated at 37°C in a humidified incubator with 5% CO_2_.

Primary cells were isolated from OC tissues obtained during in-house surgeries at the Department of Gynecology, Medical Scool, Goethe University. The study was approved by the medical ethical committee of the University Hospital Frankfurt am Main in compliance with ethical regulations. All patients provided written informed consent as approved by the responsible authority. Tissue samples were stored in DMEM (Fisher Scientific, Dreieich, Hessen, Germany) supplemented with 1% penicillin/streptomycin (Thermo Fisher Scientific, Dreieich, Hessen, Germany). Under a laminar flow hood, samples were transferred to sterile petri dishes, carefully rinsed with cold PBS (Thermo Fisher Scientific, Dreieich, Hessen, Germany), and cleared of blood, necrotic tissue and fatty inclusions. The tissue was then divided for specific applications, including 2D primary cell cultures, organoids generation, immunohistochemistry (IHC), or snap-freezing at -80℃ for later use.

For 2D Primary cell cultures, tissue samples were finely minced (1-2 mm) using a scalpel and enzymatically digested with equal volumes of 10× trypsin (Sigma-Aldrich/Merck, Darmstadt, Hessen, Germany) and collagenase-1 solution (Worthington, Lakewood, NJ, USA (1 mg/mL, filtersterilized). The digestion process was carried out in a 50 mL tube at 37 °C for 45-60 minutes, depending on tissue size and characteristics. To terminate digestion, a two-fold volume of fetal bovine serum (FBS) (Gibco/Thermo Fisher Scientific, Dreieich, Hessen, Germany) was added. The sample was then sequentially filtered through cell strainers (100 µm, 70 µm, and 40 µm), washed multiple times with PBS, and collected in a 50mL tube. The filtrate was then centrifuged at 200 × g for 5 minutes, and the supernatant was discarded. The resulting cell pellet was resuspended in primary cell growth medium containing RPMI 1640 (Fisher Scientific, Dreieich, Hessen, Germany), 2% FBS (Gibco/Thermo Fisher Scientific, Dreieich, Hessen, Germany), 1% penicillin/streptomycin (Thermo Fisher Scientific, Dreieich, Hessen, Germany), 0.01 µg/mL EGF (Sigma-Aldrich/Merck, Darmstadt, Hessen, Germany), 20 µg/mL Insulin (Merck, Darmstadt, Hessen, Germany), 0.5 µg/mL Hydrocortison (Merck, Darmstadt, Hessen, Germany), 25 ng/mL Choleratoxin (Merck, Darmstadt, Hessen, Germany), and 1 µg/mL Amphotericin B (Sigma-Aldrich/Merck, Darmstadt, Hessen, Germany)). If red blood cell contamination was significant, red blood cell lysis was performed using ammonium-chloride-potassium (ACK) buffer for 8-10 minutes at 37℃.

**Ascites preparation**
For OC patients who developed ascites, the digestion steps were omitted. Depending on the ascites volume, samples were processed by centrifugation at 200 × g for 5 minutes to concentrate cells. For low-volume samples, direct filtration through sequential cell strainers (100 µm, 70 µm, and 40 µm) was performed, followed by an additional centrifugation step at 200 × g for 5 minutes to collect cells.
Subsequently, density gradient centrifugation was applied to separate tumor cells from immune and stromal cells based on size and density. Tumor cell enrichment was further facilitated by culturing the isolated cells in low-attachment plates, promoting the preferential survival of tumor cells over non-tumor cells. Finally, ascites-derived tumor cells were transferred to a T75 cell culture flask and maintained in primary cell growth medium, as previously described.

**Organoid culture**
After the in-house procedure, tissues were immediately collected and stored in ice-cold storage medium (Dulbecco's Modified Eagle's Medium [DMEM], 1% penicillin-streptomycin; Thermo Fisher Scientific, Dreieich, Hessen, Germany). Fresh tissues were then washed three times with a wash medium composed of DMEM (500 mL), 1% penicillin-streptomycin, and 2 g of bovine serum albumin (BSA). The tissue was then chopped into tiny pieces (2-4 mm), allowed to rest on ice for two minutes, and the supernatant was removed. Depending on sample size, the tissue was then digested in 5-20 mL of digestion media at 37℃ stored for 30-40 minutes. The digestion medium contained 20 mL Advanced DMEM (Thermo Fisher Scientific, Dreieich, Hessen, Germany), 20 mg collagenase-1 (Worthington, Lakewood, NJ, USA), and 5 µmol/L Y-27632 (Biozol, Eching, Bavaria, Germany).

The sample was shaken every five to ten minutes, and the digestion progress was monitored under the microscope. Once the tissue had dissociated into single cells or small clusters, digestion was stopped by adding an equal volume of wash media. The cell suspension was filtered through a 100 µm filter, centrifuged at 200 × g for 5 minutes at 4°C, and the supernatant was discarded to remove residual collagenase. For ascites-derived OC samples, enzymatic digestion was omitted. Instead, the sample was passed through a 100 µm cell strainer, centrifuged at 200 × g for 5 minutes at 4℃, and processed as follows. If the pellet contained red blood cells, erythrocytes were lysed using ammonium-chloride-potassium buffer (red lysis buffer) composed of 0.15 mol/L NH4Cl (Sigma-Aldrich/Merck, Darmstadt, Hessen, Germany), 10 mmol/L KHCO_3_ (Sigma-Aldrich/Merck, Darmstadt, Hessen, Germany), and 0.1 mmol/L ethylenediamine-tetraacetic acid (Carl Roth, Karlsruhe, Baden-Würtemberg, Germany). Depending on pellet size, 0.5 to 1 mL red lysis buffer was added, incubated for two to three minutes at room temperature, and then washed two to three times with wash medium to remove residual lysis buffer.

After discarding the supernatant, the cell pellet was resuspended in cold wash medium and mixed with three times its volume of either Base Membrane Extract (Bio-Techne, Wiesbaden, Hessen, Germany) or Matrigel Matrix Basement Membrane (Corning/Thermo Fisher Scientific, Dreieich, Hessen, Germany). The matrix mixture was kept on ice to prevent premature solidification. To establish 3D culture, 10-20 µL droplets were quickly pipetted into a 12-well suspension plate. The plate was then inverted and incubated at 37°C with 5% CO_2_ for 20-30 minutes, allowing the matrix to solidify. Once solidified, 1 mL of growth medium was added to each well. The organoid growth medium is composed of 46.7 mL Advanced DMEM/F12 (Thermo Fisher Scientific, Dreieich, Hessen, Germany), 1% glutamin (Merck, Darmstadt, Hessen, Germany), 1% penicillin/streptomycin (Thermo Fisher Scientific, Dreieich, Hessen, Germany), 10 µmol/L Y-27632 (Biozol, Eching, Bavaria, Germany), 0.25 µmol/L A83-01 (Bio-Techne, Wiesbaden, Hessen, Germany), 1 × B27 supplement (Thermo Fisher Scientific, Dreieich, Hessen, Germany), 5 mmol/L nicotinamide (Merck, Darmstadt, Hessen, Germany), 1.25 mmol/L N-acetylcysteine (Merck, Darmstadt, Hessen, Germany), 1× N2 supplement (Thermo Fisher Scientific, Dreieich, Hessen, Germany), 1 µmol/L SB-203580 (p38i) (AdipoGen, Fuellinsdorf, Switzerland), 50 ng/mL hEGF (Peprotech/Thermo Fisher Scientific, Dreieich, Hessen, Germany), 50 ng/mL Neuregulin 1 (NRG 1) (Peprotech/Thermo Fisher Scientific, Dreieich, Hessen, Germany), 10 ng/mL HGF (Peprotech/Thermo Fisher Scientific, Dreieich, Hessen, Germany), 20 ng/mL IGF (Peprotech/Thermo Fisher Scientific, Dreieich, Hessen, Germany), 10 nmol/L β-Estradiol (Sigma-Aldrich/Merck, Darmstadt, Hessen, Germany), 200 ng/mL Primocin (InvivoGen, San Diego, CA, USA), 50 ng/mL R-Spondin (Peprotech/Thermo Fisher Scientific, Dreieich, Hessen, Germany), 100 ng/mL Noggin (Bio-Techne, Wiesbaden, Hessen, Germany). The medium was changed twice a week, and passaging was performed based on organoid size and density.

**Transfection**

mRNA transfections were performed using Lipofectamine Messenger MAX Transfection Reagent (Invitrogen) at a ratio of 1 µg IVT mRNA: 2 µL Lipofectamine Messenger MAX, following the manufacturer's instructions. For radioimmunoprecipitation assays, ovarian cancer cells were isolated and lysed in RIPA buffer (Waltham, MA, USA). The supernatant containing cytoplasmic protein was obtained after centrifugation at 15,000 ×g for 5 minutes at 4°C.

**Colony formation assay**

Two thousand cells were seeded into six-well plates. After washing, the transfected cells were cultured in fresh media for two weeks. Colonies were fixed with 70% ethanol and stained with 0.5 µg of Coomassie Brilliant Blue (Biorad, Feldkirchen, Bavaria, Germany). The number of colonies was counted, and images were captured using an AxioObserver Z1 microscope (Zeiss, Göttingen, Germany) and a ChemiDoc MP system (BioRad, Hercules, CA, USA).

**Western blot (WB) and antibodies**

Cell protein extracts were lysed using RIPA buffer (Sigma) supplemented with a complete protease inhibitor cocktail (Roche, Mannheim, Rheinland Pfalz, Germany). Sodium dodecyl sulfate-polyacrylamide gel electrophoresis (SDS-PAGE) was used to separate 25 μg of protein extracts, followed by transfer onto polyvinylidene difluoride membranes using a TransBlot Turbo Transfer System (Bio-Rad, Feldkirchen, Bavaria, Germany). Membranes were blocked with Tris-buffered saline containing 0.05% Tween® 20 (Sigma, Frankfurt, Hesen, Germany) and 2% BSA (Thermo Fisher Scientific, Dreieich, Hessen, Germany).

The following primary antibodies were used at the indicated dilutions: DAPK1 (ab109382) (1:1000; Abcam), mouse monoclonal PLK1 (F-8:sc-17783) (1:1000; Santa Cruz, Biotechnology Heidelberg, Germany), Cyclin A (H-432) (1:1000; Santa Cruz), CDK1 (sc-54) (1:1000; Santa Cruz), p53 (sc-126) (1:1000; Santa Cruz), MDM2 (sc-965) (1:1000; Santa Cruz), β-Actin (AC-15:A1978) (1:200.000; Sigma-Aldrich, Taufkirchen, Germany), Flag (M2:A8592) (1:1000; Sigma-Aldrich), Cyclin B1 (#4138) (1:1000; Millipore), pMLC-2 (#9577) (1:1000; Cell Signaling), Aurora A (#14475) (1:1000; Cell Signaling), p16 (#18769) (1:1000; Cell Signaling), Noxa (#14766) (1:1000; Cell Signaling), p14ARF (#74560) (1:1000; Cell Signaling), Beclin 1 pT119 (#ABC118) (1:1000, Millipore), p53-pS20 (#9287) (1:1000; Cell Signaling), p21 (#2947) (1:1000; Cell Signaling), PARP (#9542) (1:1000; Cell Signaling), Caspase 3 (#9665) (1:1000; Cell Signaling), Puma (#98672) (1:1000; Cell Signaling), Bax (AF820) (1:1000; R&D systems). HRP-conjugated secondary antibodies were used at a dilution of 1:5000 (GE Healthcare and Jackson Laboratory, Bar Harbor, ME, USA). ECL WB Substrate (Millipore) was used for detection.

**Cell viability and proliferation assays**

The Caspase-Glo 3/7 assay kit (Promega, Waldorf, Hessen, Germany) was utilized in compliance with the guidelines provided by the manufacturer. The measured luminescence (RLU) was presented as the mean value ± standard deviation (*n* = 3). Non-synchronized cells were treated with the test chemicals for the specified durations to assess apoptosis. For cell viability experiments, the Cell Titer-Blue® Cell Viability assay (Promega) was performed as per the manufacturer’s instructions, using fluorescence as the readout at excitation and emission wavelengths of 562 nm and 615 nm. Statistical significance between data groups was determined using the Student's two-tailed test (**P* ≤ 0.05; ***P* ≤ 0.01; ****P* ≤ 0.001).

Cell proliferation assays were carried out using the Cell Titer-Blue® Cell Viability assay (Promega). Briefly, cells seeded in 96-well plates were transfected with DAPK1-mRNA or control mRNA. Afterwards, a Victor X4 Multilabel Counter (Victor X4, PerkinElmer, Rodgau, Hessen, Germany) was used to measure the fluorescence at designated time points.

**Animal experiments**

All animal studies were approved by the Regierungspräsidium Darmstadt and conducted under the supervision of the Division of Laboratory Animal Medicine at Georg-Speyer-Haus and Goethe University's Medical School. 6–8 weeks old female nude mice (body weight: 25-30 g) were purchased from Charles River Laboratories (Sulzfeld, Bavaria, Germany) and housed under a 12-hour light/dark cycle. 70% ethanol was used to sterilize the injection site. Using forceps to carefully separate the skin, 2 × 10^6^ stable Luc-expressing OVCAR-8 cells were intraperitoneally injected. 1-day post-injection, the mice received two weekly intraperitoneal injections of either $\Delta DAPK1$-mRNA (0.16 mg/kg) or a control for a total duration of three weeks.

***In vivo* imaging system (IVIS)**

Bioluminescence imaging (BLI) using IVIS was employed to monitor tumor progression and dissemination. The animals were anesthetized using isoflurane, and 100 µL of luciferin (Promega, Waldorf, Hessen, Germany) was administered subcutaneously 15 minutes prior to bioluminescence measurement. Light signal emission was detected using an IVIS Lumina II Multispectral Imaging System (PerkinElmer, Rodgau, Hessen, Germany). The first measurement was taken on the day of cell injection, followed by a second imaging session seven days later. Subsequent imaging was performed once per week.

**Tissue processing for anatomical and histological examination**

Mice were sedated with isoflurane gas, injected with 100 µL luciferin, and maintained in an isoflurane chamber for 15 minutes to allow luciferin distribution in the organs for a gross anatomical evaluation of control and treated mice. Following euthanasia by cervical dislocation, the animals were positioned in dorsal recumbency and secured with needle pins. The skin and body walls were incised along the linea alba, and photos of internal organs were taken. The liver, kidneys, uterus and ovaries, spleen, omentum, small and large intestines, lungs, and peritoneal surfaces were then examined macroscopically under a microscope. Any significant morphological alterations in the organs were documented.

Tissues and organs were carefully excised and placed in Petri dishes (small and large intestines) or six-well plates (all other organs) to keep them hydrated. Following IVIS imaging, tissues were either snap-frozen on dry ice for molecular analysis or fixed in PBS-buffered formalin for histological and immunohistochemical investigations.

Whole ovaries, including the bursa ovarica, along with samples from the liver, spleen, kidneys, and lungs, were dissected. Intestinal tissues were prepared following the Swiss roll technique for optimal histological examination. Specimens were fixed in 10% neutral-buffered formalin for 48 hours at room temperature, followed by repeated PBS washings to remove residual formalin. The tissues were then dehydrated in graded ethanol, cleared in xylene, and embedded in paraffin. Serial 5 µm sections were cut from paraffin blocks and stained with hematoxylin and eosin (H&E) for microscopic examination of overall histology and potential malignancies.

**Statistical methods**

All experiments were performed in at least triplicates. Statistical analyses were conducted using Microsoft Excel and GraphPad Prism. For paired comparisons, experimental groups were analyzed relative to their respective controls. Statistical significance between two groups was assessed using the Student’s t-test and Wilcoxon test, unless otherwise specified. A *P* value < 0.05 was considered statistically different. Significant differences are indicated by asterisks (**P* ≤ 0.05, ***P* ≤ 0.01, and ****P* ≤ 0.001).

**Supplementary Figure Legends**


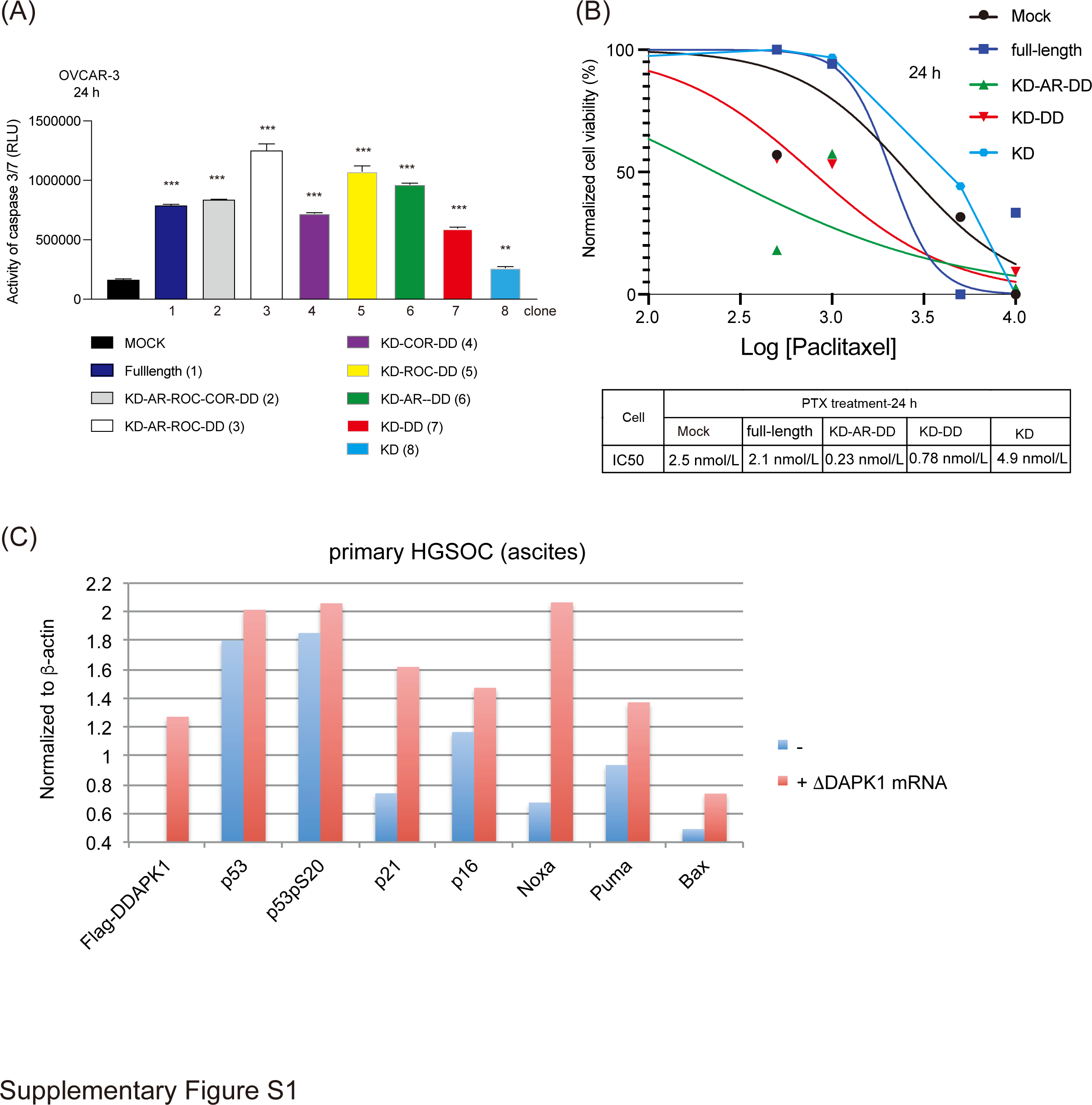


**Supplementary Figure S1. Evaluation of truncated DAPK1 clones for apoptosis-inducing potential in ovarian cancer cells.**

(A) Caspase-Glo 3/7 assay was used to assess Caspase-3/7 activity of OVCAR-3 cells transfected with recombinant full-length and truncated DAPK1-expressing vectors. ***P* < 0.01, and ****P* < 0.001, Student's unpaired two-tailed t-test.

(B) DAPK1 functional domains, including the kinase domain (KD), Ca^2+^/CaM autoregulatory domain (CM), Ankyrin Repeats (AR), Ras of complex protein (ROC) - C-terminal of ROC (COR) domain, and death domain (DD). Cells transfected with full-length DAPK1 or truncated sub-clones (KD-AR-ROC-COR-DD, KD-AR-ROC-DD, KD-ROC-DD) were treated with increasing concentrations of Paclitaxel, and cell proliferation was measured.

(C) Densitometric analysis and quantification of key cell cycle and apoptotic markers following IVT-mRNA DAPK1 expression.

Abbreviations: KD-AR-ROC-COR-DD, DAPK1 kinase domain-ankyrin repeats-ras of complex-cystathionine β-synthase domains-Death Domain; KD-AR-ROC-DD, DAPK1 kinase domain-ankyrin repeats- domains-Death Domain; KD-COR-DD, DAPK1 kinase domain-cystathionine β-synthase domains-death domain; KD-ROC-DD, DAPK1 kinase domain-ras of complex-cystathionine β-synthase domains-Death Domain; KD-AR-DD, DAPK1 kinase domain-ankyrin repeats-death domain; KD-DD, DAPK1 kinase domain-death domain; KD, DAKP1 kinase domain; p53, tumor protein p53; p53pS20, p53 phosphorylated at Ser20; p21 (CIP1/WAF1), cyclin-dependent kinase inhibitor p21; p16 (INK4a), cyclin-dependent kinase inhibitor that inhibits CDK4/6; Noxa, pro-apoptotic protein regulated by p53; Puma, p53 upregulated modulator of apoptosis; Bax, Bcl-2-associated X protein.

**Supplementary Figure S2.** **Reactivation of DAPK1 functions in HGSOC cell lines by ∆DAPK1-IVT mRNA.**(A) OVCAR-8 cells were transfected with increasing concentrations of ΔDAPK1-IVT mRNA. On day 7, representative images illustrate a dose-dependent reduction in 2D colony formation (left panel). A bar graph quantifies the dose-dependent distribution of colonies (right panel, *n* = 3). Student's unpaired two-tailed t-test, **P* < 0.05, ***P* < 0.01, ****P* < 0.001.

(B) OVCAR-8 cells were transfected with increasing concentrations of ΔDAPK1-IVT mRNA. Western blot analysis of cell lysates was performed using Flag, pMLC2, phospho-p53 (pS20), PLK1, Cyclin A, Cyclin B1, Aurora A, CDK1, PARP, Caspase 3, phospho-Beclin 1 (pT119), p14ARF, and β-Actin antibodies (upper panel). Densitometric analysis and quantification of key cell cycle, apoptotic, and autophagy markers following IVT-mRNA DAPK1 expression (lower Panel).

(C) A Caspase-Glo 3/7 assay was used to measure DAPK1-IVT mRNA dose-dependent Caspase-3/7 activity. **P* < 0.05, ***P* < 0.01, and ****P* < 0.001, Student's unpaired two-tailed t-test.

Abbreviations: ∆DAPK1-mRNA, truncated form of DAPK1 (KD-AR-DD); p53, tumor protein p53; p53pS20, p53 phosphorylated at Ser20; PLK1, polo-like kinase 1; CDK1, cyclin-dependent kinase 1; PARP, Poly (ADP-ribose) polymerase; Casp.3, Caspase 3, pBeclin, phospho-Beclin; LC3A/B-I, microtubule-associated proteins 1A/1B light chain 3A/B-I; LC3A/B- II, microtubule-associated proteins 1A/1B light chain 3A/B-II; p14ARF, alternate reading frame protein; MLC2, myosin Light chain 2.


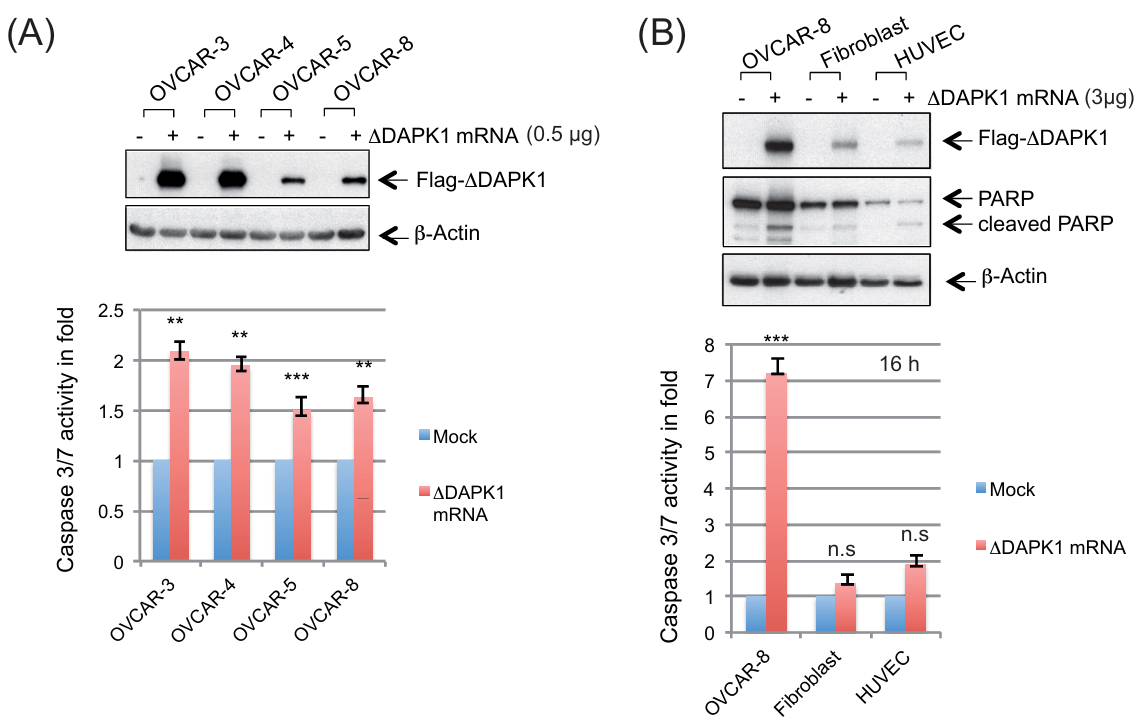


**Supplementary Figure S3. Evaluation of ∆DAPK1-IVT mRNA transfection effects on protein expression and caspase-3/7 activity in ovarian cancer cells.**
(A) Ovarian cancer cells lines were transfected with ∆DAPK1-IVT mRNA. Cell lysates were analyzed by Western blot using Flag, and β-Actin antibodies (upper panel). Caspase-3/7 activity in response to ∆DAPK1-IVT mRNA treatment was assessed using a Caspase-Glo 3/7 assay (lower panel). ^**^*P* < 0.01, ^***^*P* < 0.001, Student’s unpaired two-tailed t-test.

(B) OVCAR-8 cells and primary human cells were transfected with ∆DAPK1-IVT mRNA. Cell lysates were analyzed by Western blot using Flag, PARP, and β-Actin antibodies (upper panel). Caspase-3/7 activity in response to ∆DAPK1-IVT mRNA treatment was assessed using a Caspase-Glo 3/7 assay (lower panel). ^***^*P* < 0.001, Student’s unpaired two-tailed t-test.

Abbreviations: ∆DAPK1-mRNA, truncated form of DAPK1 (KD-AR-DD); PARP, Poly (ADP-ribose) polymerase.


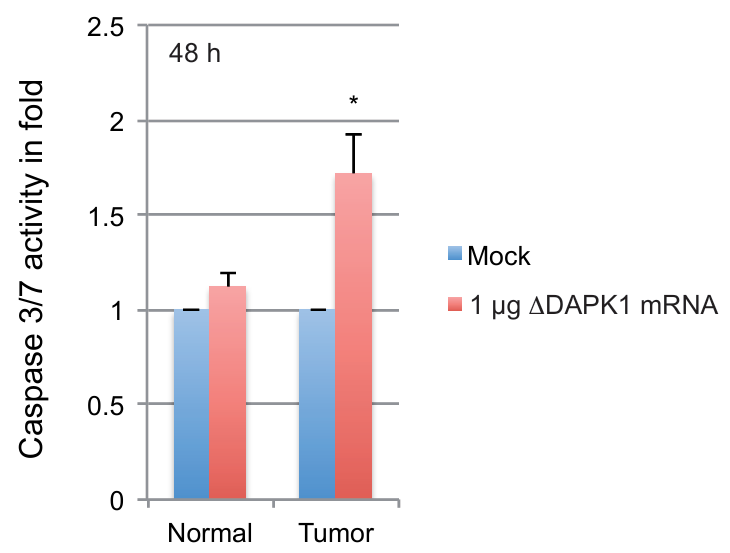


**Supplementary Figure S4.** **Effects of DAPK1 reactivation on the survival of primary HGSOC cells.**

Patient-derived primary human HGSOC cells (tumor, normal) were transfected with 1 μg ∆DAPK1-IVT mRNA. Caspase-Glo 3/7 assays were performed to assess Caspase-3/7 activity 48 hours post-transfection. * *P* < 0.05, Student's unpaired two-tailed t-test.

Abbreviations: ∆DAPK1-mRNA, truncated form of DAPK1 (KD-AR-DD).


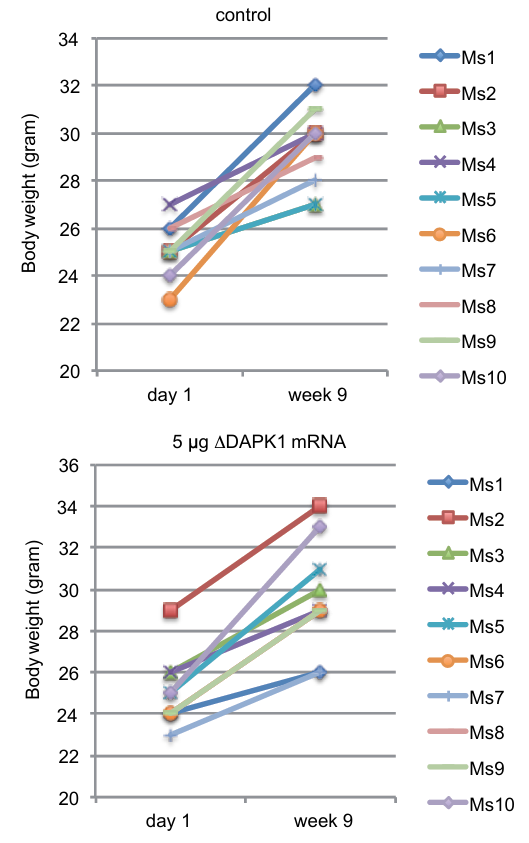


**Supplementary Figure S5. In a xenograft mouse model, intraperitoneal injection of OVCAR-8 cells followed by intraperitoneal liposomal ∆DAPK1-IVT mRNA therapy inhibits tumor growth and metastatic spread.**

Body weight was monitored throughout the observation period.

Abbreviations: ∆DAPK1-mRNA, truncated form of DAPK1 (KD-AR-DD).


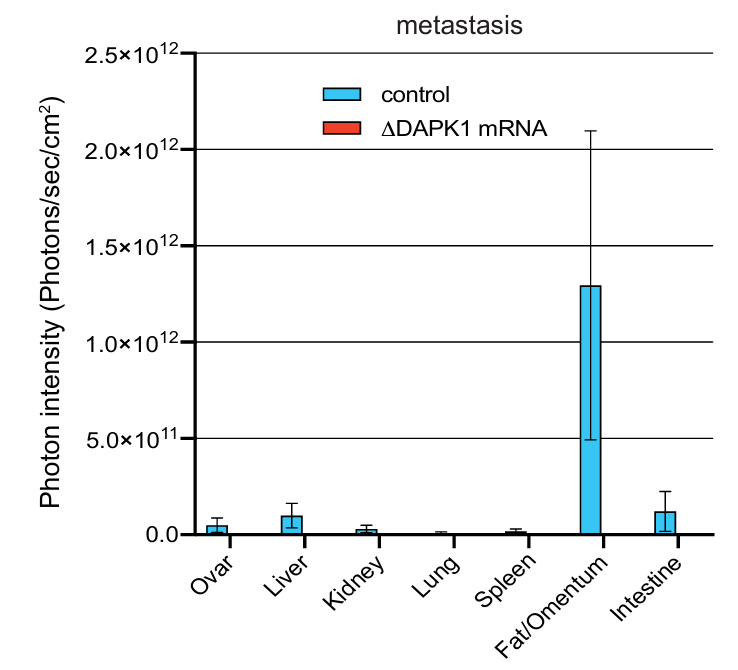


**Supplementary Figure S6.** **Bioluminescence imaging analysis of metastatic spread in excised organs from mice treated with ∆DAPK1-IVT mRNA compared to the control. (*n*= 5 mice per condition).**

Abbreviations: ∆DAPK1-mRNA, truncated form of DAPK1 (KD-AR-DD).
